# Lateral lower thoracic lipomyelomeningocele: a case report

**DOI:** 10.4076/1757-1626-2-8122

**Published:** 2009-07-01

**Authors:** Fatih Serhat Erol, Necati Ucler, Huseyin Yakar

**Affiliations:** Department of Neurosurgery Elazig, Firat UniversitesiTip Fakultesi, Firat Tip Merkezi, Beyin Cerrahi Klinigi, 23200, ElazigTurkey

## Abstract

**Introduction:**

Lipomyelomeningocele is a form of the spina bifida occulta, which show differentiation from spina bifida aperta by pathogenesis. Laterally located thoracal lipomyelomeningocele is very rare congenital spinal pathology. The clinical presentation may be soft tissue mass, sensorial-motor loss and/or bladder dysfunction.

**Case presentation:**

Here we presented a 10-month-old girl patient harboring lipomyelomeningocele in left lateral side of the lower thoracal region.

**Conclusions:**

We have not found the entity of lateral lower thoracal lipomyelomeningocele and especially as large as this size mass at ages of our patient in the literature. We operated our patient for the possible emerging complications and purposed prevention of these complications with this intervention.

## Introduction

LMMC (or lipoma of the conus medullaris) the most commonly fatty mass occurring along or within the spine. LMMC is a form of occult spinal dysraphism in which a subcutaneous fibrofatty mass traverses the lumbodorsal fascia, causes a spinal laminar defect, displaces the duramatter, and infiltrates and tethers the spinal cord [1-3].

LMMC is the commonest cause of congenital tethering and causes neurological deterioration due to the conus medullaris and root ischemia [3-5]. We have not found the entity of lateral lower thoracal LMMC and especially as large as this size mass at ages of our patient in the literature.

Here we presented a 10-month-old girl patient harboring LMMC, which was under skin and extending to spinal canal in left lateral lower thoracal (Th) region without neurological deficit and urinary dysfunction.

## Case presentation

**Figure 1.**
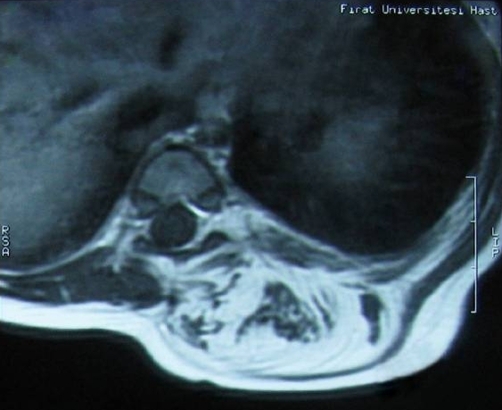
Axial MRI view demonsrating the mass which belonging to lipomyelomeningocele was located to left lateral lower thoracic region. As seen from this axial view, the mass was extending from the paraspinal region to spinal canal and almost deplacing the spinal cord.

**Figure 2.**
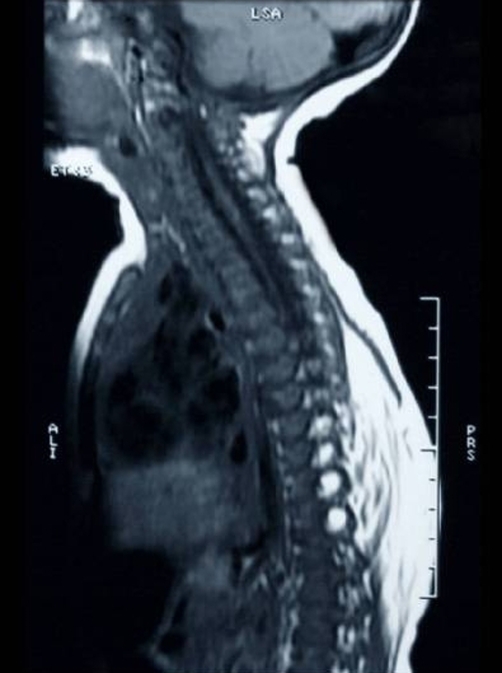
Sagital MRI demonstrating involvement of the lower thoracic region by LMMC.

The Turkish 10-month-year old girl who born with normal spontaneous vaginal delivery admitted to our out-patient clinic for progressively swelling mass. This mass was approximately 12 × 8 cm size over left lateral lower thoracal region under skin after birth for approximately 3 months. Her mother took periconceptual folic acid and there was no pathological finding in her gynecological controls. Vaccination history was satisfactory. Such this congenital anomaly learned from family history was not seen before. On examination, there was no remarkable finding in the patient, except cosmetic swelling in the left lateral side of the lower thoracal region. From the patient's history, no neurological retardation or abnormality were determined by her family and pediatrician. On our clinical examination, no neurological deficit was found and urinary dysfunction tests were negative. Magnetic resonance imaging (MRI) studies showed LMMC that was located at the level between left Th9-Th12 paraspinal region, including muscle fibers, expanding spinal foramina at these levels, extending to spinal canal and slightly deplacing the canal to right lateral, and the greatest diameters of LMMC were 5 × 3.5 cm and 12 × 3 cm at axial images and sagittal images in fat-suppressed sequences, respectively (Figures 1,2).

The patient was operated under general anesthesia in prone position, after subcutaneous dissections at left lateral lower thoracal region, we met fatty tissue which was extending to the 9, 10, 11, 12, and following and dissecting these pathologic fatty formations, we reached the spinal canal through the small dural defect at base and then micro-surgically excised these adhered lesions from spinal canal and the lipomyelomeningocele was corrected without any complication. The excision material that sent to pathology laboratory during surgery reported as LMMC by the laboratory.

## Discussion

Related to LMMC, the mechanism was proposed by Mc Lone et.al. Cutaneous and neural ectoderm separation occurs after neural tube closed normally but in this mechanism of LMMC, this separation occurs before neural tube closing and paraxial mesenchymal tissue prevents closure of open neural tube and causes a segmental myeloschisis by effect of the lateral somatic mesoderm that is continuous to grow [6]. Marine-Padilla proposed an other theory. According to them, the paraxial mesoderm which was accepted as main stimulant to embryologic layers was insufficient [7]. Also the theory in which excessive neural tissue growth preventing neural cleft fusion in spina bifida region [8] and an autosomal inheritance were also suggested for LMMC [5].

LMMC are lipomas that are tightly attached to the dorsal surface of a neural placode and extend dorsally through a spina bifida to be continuous with the subcutaneous fat [1-3]. LMMC rate has been estimated to be 2.5 per 10000 births [9]. In general, lipomas located subcutaneously have benign behavior and cause no problem except cosmetical matter but according to their localization, sometimes may cause pressure symptoms. In our first examination, the lesion was subcutaneously located to left lateral lower thoracal region, palpated as not tough mass and causing no pressure symptoms in our patient. Despite the patient had no complaint except cosmetical matter but we got suspicious about any congenital pathology and asked radiologic imaginations which revealed the subcutaneous lesion extending spinal canal through thoracal fassia, lamina and than piamatter. The above findings were confirmed on surgery.

Majority of such these lesions are considered to be subcutaneously located benign mass, especially for pediatric age group of patients. If the benign mass was located to subcutaneously patient’s posterior region, even if lateral region, LMMC must be held in our mind for differential diagnosis of posterolateral located masses. Necessary diagnostic tools must be used and prompt referral to neurosurgery department for possible emerging complications is advantageous for patients with this type lesion.

LMMC is the most common cause of tethered cord [3-5] but related to which neurological deterioration or urinary dysfunction were not found in our patient. Because the natural progression of LMMC is causing neurologic deficits, and/or urological disturbances, the prophylactic surgery is recommended for these lesions by some authors [1,10]. Treatment of LMMC is like other lesions and we operated the patient just as other lesions, but localization and size of the lesion in our case was especially important because approach to this type lesion must be different in context of thoracal vs. abdominal complications, characteristics of spinal canal, and neurological complications When thoracal spinal surgery is considered, at least these features must be in mind.

Rarely growth of spinal lipomas may be found on sequential imaging studies, and lipomas have occasionally recurred after resection and lipomas will typically enlarge with age in proportion to body growth so that significant weight gain, particularly if associated with obesity, may contribute to recurrence of spinal lipomas [11].

The lateral lower thoracic LMMC may be considered as a variant of LMMC, which is located subcutaneously lateral lower thoracal region and then extending to spinal canal where it may cause neurological pathologies at birth or with advancing age and/or weight gain. In our patient, symptoms or radiologic findings of tethered cord were not detected for 15 months follow-up.

## Conclusions

Here we presented lateral lower thoracic LMMC, which is needed detailed researches for treatment, follow-up and results of this localization of LMMC and at present, these parameters may be modified to LMMC parameters of other vertebra localization.

## Abbreviations

LMMC: Lipomyelomeningocele
MRI: Magnetic resonance imaging
Th: Thoracic

## References

[bib-001] Blount JP, Elton S (2001). Spinal lipomas. Neurosurg Focus.

[bib-002] Naidich TP, Mclone DG, Mutluer S (1983). A new understanding of dorsal dysraphism with lipoma: radiological evaluation and surgical correction. AJR Am J Roentgenol.

[bib-003] Oakes W, Wilkins RH, Rengachary SS (1991). Management of spinal cord lipomas and lipomyelomeningoceles. Neurosurgery update.

[bib-004] Cochrane DD, Finley C, Kestle J, Steinbok P (2000). The patterns of late deterioration in patients with transitional lipomyelomeningocele. Eur J Pediatr Surg.

[bib-005] Kannu P, Furneaux C, Aftimos S (2005). Familial lipomyelomeningocele: A further report. Am J Med Genet A.

[bib-006] Mclone DG, Suwa J, Collins JA, Poznanski S, Knepper PA (1983). Neurulation: biochemical and morphological studies on primary and secondary neural tube. Concepts Pediatr Neurosurg.

[bib-007] Marin-Padilla M, Holtzmann RNN, Stein BM (1985). The tethered cord sendrome: Developmental considirations. The tethered spinal cord.

[bib-008] Anderson H, Carlsson CA, Rosengren K (1967). A radiological study of the central canal in myelomeningocele. Dev Med Child Neurol Suppl.

[bib-009] McLone DG, Thompson DN, McLone DG (2001). Lipomas of the spine. Pediatric neurosurgery: surgery of the developing nervous system.

[bib-010] Wu H, Kogan BA, Baskin LS, Edwards MSB (1998). Long-term benefits of early neurosurgery for lipomyelomeningocele. J Urol.

[bib-011] Kanev PM, Berger MS, Youmans JR (1996). Lipomyelomeningocele and myelocystocele. Neurological surgery.

